# Preclinical pharmacology of glucosylceramide synthase inhibitor venglustat in a GBA-related synucleinopathy model

**DOI:** 10.1038/s41598-021-00404-5

**Published:** 2021-10-22

**Authors:** Catherine Viel, Jennifer Clarke, Can Kayatekin, Amy M. Richards, Ming Sum R. Chiang, Hyejung Park, Bing Wang, Lamya S. Shihabuddin, S. Pablo Sardi

**Affiliations:** 1 Rare and Neurologic Diseases, Sanofi, 49 New York Avenue, Framingham, MA 01701 USA; 2 Genomic Medicine Unit, Sanofi, Framingham, MA 01701 USA; 3 US Early Development for Synthetics Platform, Sanofi, Waltham, MA 02451 USA; 4 5AM Ventures, Boston, MA 02116 USA

**Keywords:** Neuroscience, Diseases of the nervous system, Drug discovery, Pharmacology

## Abstract

Mutations in *GBA*, the gene encoding the lysosomal enzyme glucocerebrosidase (GCase), represent the greatest genetic risk factor for developing synucleinopathies including Parkinson’s disease (PD). Additionally, PD patients harboring a mutant *GBA* allele present with an earlier disease onset and an accelerated disease progression of both motor and non-motor symptoms. Preclinical studies in mouse models of synucleinopathy suggest that modulation of the sphingolipid metabolism pathway via inhibition of glucosylceramide synthase (GCS) using a CNS-penetrant small molecule may be a potential treatment for synucleinopathies. Here, we aim to alleviate the lipid storage burden by inhibiting the de novo synthesis of the primary glycosphingolipid substrate of GCase, glucosylceramide (GlcCer). We have previously shown that systemic GCS inhibition reduced GlcCer and glucosylsphingosine (GlcSph) accumulation, slowed α-synuclein buildup in the hippocampus, and improved cognitive deficits. Here, we studied the efficacy of a brain-penetrant clinical candidate GCS inhibitor, venglustat, in mouse models of *GBA*-related synucleinopathy, including a heterozygous *Gba* mouse model which more closely replicates the typical *GBA*-PD patient genotype. Collectively, these data support the rationale for modulation of GCase-related sphingolipid metabolism as a therapeutic strategy for treating *GBA-*related synucleinopathies.

## Introduction

Synucleinopathies, including Parkinson’s disease (PD) and dementia with Lewy bodies (DLB), are a group of progressive neurodegenerative diseases characterized histopathologically by the aberrant α-synuclein accumulation throughout the central nervous system (CNS)^1,2^. Mutations in the *GBA* gene are a known genetic risk factor for developing a synucleinopathy^3,4^. *GBA* encodes for the lysosomal hydrolase glucocerebrosidase (GCase), which catabolizes the glycosphingolipids glucosylceramide (GlcCer) and glucosylsphingosine (GlcSph) into ceramide and sphingosine, respectively. Homozygous and compound heterozygous *GBA* mutations cause the rare lysosomal storage disorder Gaucher disease, which is characterized by reduced GCase activity and subsequent lysosomal GlcCer and GlcSph accumulation in the brain and viscera^5^.

The link between synucleinopathies and *GBA* mutations is supported by a growing body of data highlighting that Gaucher disease patients and heterozygous *GBA* mutation carriers are at an increased risk for developing PD and dementia with Lewy bodies. Additionally, PD patients carrying *GBA* mutations (GBA-PD) present with earlier onset of disease and faster cognitive decline compared to non-*GBA* mutation carriers^6–12^*.* Interestingly, sporadic PD patients can also present with decreased GCase activity in cerebrospinal fluid (CSF) and brain^13–18^. Furthermore, age-related and progressive reduction in GCase activity has been demonstrated in the substantia nigra and putamen of post-mortem brain homogenates from unaffected individuals^19^. Reduced expression of GCase protein has been shown in several cortical areas of the brain that are impacted by α-synuclein deposition and are associated with reduced lysosomal chaperone-mediated autophagy and ceramide levels^18,20^. These observations are supported by data from numerous preclinical studies performed in in vitro and in mouse models of Gaucher disease and synucleinopathy, which highlight the link between diminished GCase activity and α-synuclein aggregation^21–25^. Though the precise mechanisms underlying the role of *GBA* mutations in disease pathogenesis remain unknown, these genetic and biochemical observations support the hypothesis that loss of GCase activity and the resulting sphingolipid dyshomeostasis are critical contributors to synucleinopathies.

Importantly, a number of therapeutic interventions targeting the *GBA* pathway are entering the clinical arena. These therapeutic interventions range from the use of GCase chaperones or direct *GBA* gene augmentation to increase GCase activity, to modulation of the levels of *GBA-*related glycosphingolipids^26^. These studies are supported by robust preclinical studies in models of lysosomal storage disorders and synucleinopathies demonstrating that perturbation of the de novo glycosphingolipid synthesis pathway at specific nodes can deliver beneficial effects on associated disease pathology. For example, adeno-associated virus (AAV)-mediated overexpression of human *GBA* in the CNS of Gaucher-related synucleinopathy mice (*Gba*^*D409V/D409V*^) reduced the aberrant accumulation of GlcSph and slowed the accumulation of protein inclusions in the CNS, notably reducing insoluble α-synuclein^23^. Perhaps most importantly, the cognitive deficit displayed in this model was corrected following AAV-mediated human GCase overexpression in the brain, suggesting that select pathology may be reversible^23^. Similar benefits have been observed following the correction of glycosphingolipid substrate imbalance via substrate reduction therapy. Of note, pharmacological inhibitors of glucosylceramide synthase (GCS), the enzyme which glycosylates ceramide to produce GlcCer, have been shown to reduce glycosphingolipid accumulation and improve associated pathologies in neuropathic Gaucher disease mouse models^27–29^. Furthermore, we have previously demonstrated that inhibition of GCS can reduce glycosphingolipid levels and pathological α-synuclein inclusions in the brain and ameliorate the associated cognitive deficits in mouse models of synucleinopathy^27^. Independent groups have produced similar beneficial results in vitro through inhibition of upstream lysosomal hydrolases involved in the lipid metabolic pathway^30^. Collectively, these data suggest that restoration of lipid homeostasis via modulation of the GCase-related pathway may confer significant benefits in improving disease pathology in synucleinopathy patients.

Here, we evaluated GCS inhibition as a therapeutic strategy for *GBA*-related synucleinopathies by extending our analysis to a heterozygous *Gba* mouse model (*Gba*^*D409V/WT*^, *GBA*-related synucleinopathy mice). This mouse model carries one mutant copy of the *Gba* gene, similar to GBA-PD patients, with a point mutation in the *Gba* gene locus resulting in diminished lysosomal GCase activity in the viscera and brain. While this model lacks a cognitive deficit and exhibits reduced α-synuclein aggregation and glycosphingolipid burden in the CNS compared to homozygous *Gba* mice (*Gba*^*D409V/D409V*^, Gaucher-related synucleinopathy mice), chronic administration of an orally available GCS inhibitor, GZ667161, resulted in a decrease in brain glycosphingolipids and a numerical decrease in proteinaceous aggregates in the CNS of *Gba*^*D409V/WT*^ mice, though it did not achieve statistical significance. We then opted to evaluate our clinical candidate compound, venglustat, in the more severe *Gba* mouse model, *Gba*^*D409V/D409V*^. Akin to what we previously reported in Gaucher-related synucleinopathy mice treated with a tool compound (GZ667161)^27^, venglustat administration significantly reduced the accumulation of glycosphingolipids and pathological α-synuclein aggregates in the CNS and, importantly, ameliorated the associated memory deficit. These data provide preclinical evidence of efficacy using the clinical candidate GCS inhibitor, venglustat, in slowing disease progression in a mouse model of *GBA*-related synucleinopathy.

## Results

### Sustained GCS inhibition reduced glycosphingolipid levels in the plasma and brain of GBA-related synucleinopathy mice

**Figure 1:**
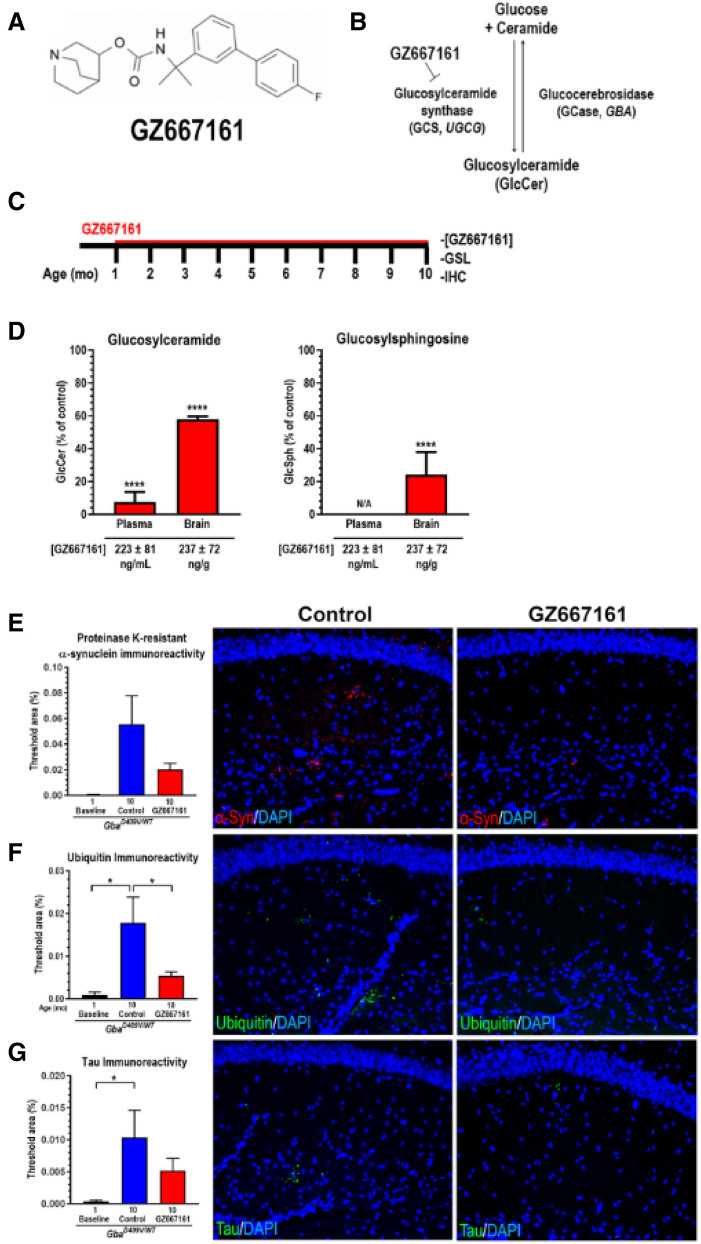
CNS inhibition of glucosylceramide synthase (GCS) by tool compound GZ667161 reduced glycosphingolipids and the accumulation of pathological CNS aggregates in a mouse model of *GBA*-related synucleinopathy. (**A**) Tool compound GZ667161 structure. (**B**) Schematic of GCS inhibition by GZ667161. (**C**) Timeline of GZ667161 administration. *GBA-*related synucleinopathy mice (*Gba*^*D409V/WT*^) were fed GZ667161 as described in the Methods and untreated littermates were fed a control diet without the small molecule compound. (**D**) GZ667161 reduces brain and plasma GlcCer and brain glucosylsphingosine (GlcSph), an accumulating glycosphingolipid in this mouse model, following 9 months of treatment. Plasma GlcSph levels are not shown as they are below the lower limit of quantification. GZ667161 concentration in plasma and brain are shown below the x-axis. Results are represented as mean ± SEM, *N* ≥ 15. Unpaired *t*-test; ****p < 0.0001. (**E**–**G**) Brain sections from *Gba*^*D409V/WT*^ mice were immunostained for proteinase K-resistant α-synuclein (**E**), ubiquitin (**F**), and tau (**G**) aggregates. GCS inhibition by GZ667161 results in an insignificant numerical decrease in proteinase K-resistant α-synuclein and tau hippocampal aggregates and a significant reduction in ubiquitin aggregates compared to littermate controls. The representative images (*right*) show proteinase K-resistant α-synuclein immunoreactivity (**E**; red), ubiquitin immunoreactivity (**F**; green), and tau (**G**; green) of control and GZ667161 treated *Gba*^*D409V/WT*^ mice at 10 months of age. DAPI-stained cell nuclei are shown in blue. One-way ANOVA followed by Tukey’s post-hoc analysis. All data represent the mean ± SEM, with *N* = 10–15 per group; *p < 0.05.

Given that *GBA* haploinsufficiency significantly increases the risk for developing a synucleinopathy, we evaluated the effects of GCS inhibition on disease pathology in a *GBA*-related synucleinopathy mouse model (*Gba*^*D409V/WT*^). *Gba*^*D409V/WT*^ mice carry one mutant copy of the murine *Gba* gene and exhibit multiple features of synucleinopathy, including accumulation of α-synuclein inclusions and reduced GCase activity in the CNS^22^. To evaluate the effects of sustained GCS inhibition on disease pathologies, we performed a long-term GCS inhibitor administration study, in which *Gba*^*D409V/WT*^ mice were fed GZ667161 (Fig. 1A,B), a brain-penetrant GCS inhibitor and an analog of the clinical candidate compound venglustat with similar GCS inhibitory activity (Suppl. Table 1). *Gba*^*D409V/WT*^ mice were administered GZ667161 in pelleted diet (0.033% wt/wt) starting at 4 weeks of age until they were 10 months old (Fig. 1C). Control *Gba*^*D409V/WT*^ mice were given vehicle rodent chow for the same duration. At the time of sacrifice, mean drug exposures were 223 ± 81 ng of drug per mL of plasma and 237 ± 72 ng drug per gram of wet brain homogenate (Fig. 1D). GlcCer levels were significantly reduced in *Gba*^*D409V/WT*^ mice following GZ667161 administration. After 9 months of treatment, GlcCer levels in plasma were reduced to 8 ± 6% relative to controls (*p* < 0.0001) while GlcCer levels in the brain were reduced to 58 ± 2% relative to controls (*p* < 0.0001; Fig. 1D). The levels of GlcSph in the brains of control and treated mice were below the limit of quantitation and were therefore not compared (Suppl. Table 2)^22^. Together, these data demonstrated that sustained GCS inhibition effectively reduced GCase-related glycosphingolipid substrates in the CNS of *GBA*-related synucleinopathy mice.

### Long-term GCS inhibition reduced hippocampal protein aggregates in GBA-related synucleinopathy mice

Aberrant protein misfolding has been implicated in synucleinopathies including PD and DLB^31^, a pathological phenotype which was recapitulated in mutant *Gba* mouse models. To that effect, we have previously shown progressive CNS accumulation of protein inclusions composed of tau, ubiquitin, and proteinase K-resistant α-synuclein in Gaucher-related synucleinopathy mice (*Gba*^*D409V/D409V*^)^22^. These protein inclusions were reduced following long-term GCS inhibition^27^. Here, we applied the same therapeutic agent to the *GBA*-related synucleinopathy mice (*Gba*^*D409V/WT*^). While untreated *Gba*^*D409V/WT*^ mice accumulated some protein aggregates in the brain, mice treated for 9 months with GZ667161 exhibited a decrease in proteinase K-resistant α-synuclein, tau, and ubiquitin aggregates (*p* = 0.2, 0.3, and 0.03, respectively; Fig. 1E–G). However, due to the modest levels of inclusions that accumulated in these mice even by 10 months of age, the reduction in protein aggregates following GZ667161 treatment was only statistically significant for the ubiquitin aggregates (*p* = 0.03). Regardless, the beneficial outcomes achieved here further support the rationale for modulating sphingolipid metabolism homeostasis as a means of relieving aberrant protein aggregation in the CNS.

### Venglustat, a brain-penetrant GCS inhibitor, reduced glucosylceramide in mouse models of GBA- and Gaucher-related synucleinopathy

Following our evaluation of GCS inhibitors using GZ667161 to mediate disease pathology in *GBA*-related synucleinopathy mice (*Gba*^*D409V/WT*^), we sought to evaluate the efficacy of the clinical candidate GCS inhibitor compound, venglustat. Venglustat is currently in development as a novel GCS inhibitor in multiple phase 2 and phase 3 clinical trials for several diseases in which the sphingolipid metabolic pathway at the node of GlcCer/GCS is pathophysiologically implicated. Based on results from phase 1 clinical studies and nonclinical studies evaluating the safety and tolerability, pharmacokinetic, and pharmacodynamic profiles, oral administration of venglustat was generally well-tolerated with minimal side effects and off-target pharmacological effects (Suppl. Tables 3 and 4)^32^.

**Figure 2:**
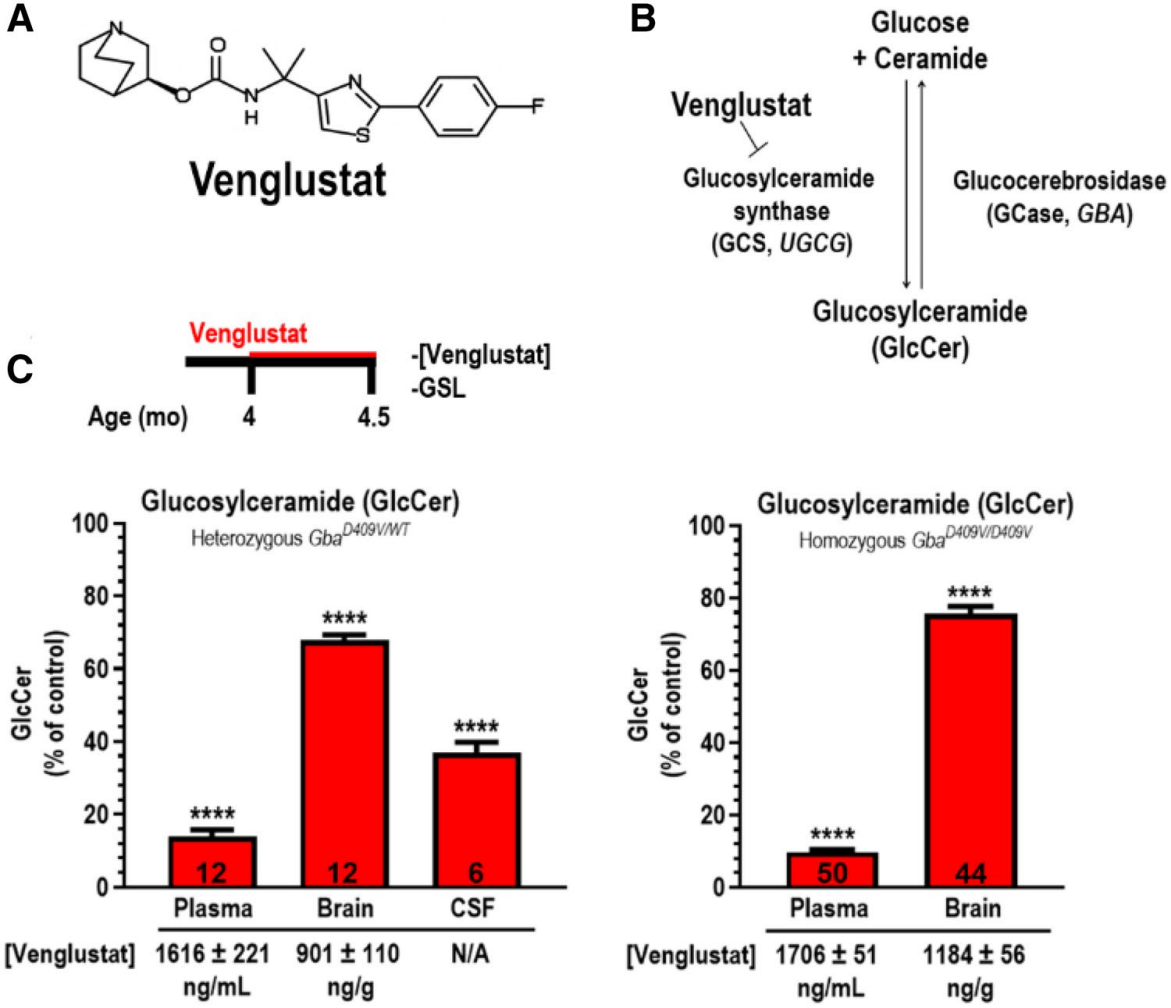
CNS inhibition of GCS by venglustat reduced glycosphingolipids in mouse models of *GBA*- and Gaucher-related synucleinopathy. (**A**) Venglustat structure. (**B**) Schematic of GCS inhibition by venglustat. (**C**) Time of venglustat administration and confirmation of target engagement following venglustat administration. Four-month-old mice were orally administered venglustat, as described in the Methods, for two weeks prior to euthanasia and tissue collection. Littermates were fed the same rodent chow without the small molecule drug. Venglustat administration in *GBA*-related synucleinopathy mice (*Gba*^*D409V/WT*^) significantly reduced glucosylceramide (GlcCer) in the plasma, brain, and cerebrospinal fluid (CSF) compared to untreated control mice (*N* = 6–12; *left*). Venglustat also significantly reduces plasma and brain GlcCer in treated Gaucher-related synucleinopathy mice (*Gba*^*D409V/D409V*^) compared to controls (*N* = 44–50; *right*), confirming target engagement. Limited CSF volumes precluded us from measuring GlcCer in Gaucher-related synucleinopathy mice. Plasma and brain exposure to venglustat was confirmed in both mouse models and is shown below the x-axes. CSF exposure was not evaluated in *GBA*-related synucleinopathy mice due to limited sample volumes. Drug concentration in CSF was 56 ± 3 ng/mL (*N* = 36; data not shown) in *Gba*^*D409V/D409V*^ mice. Results are represented as the mean ± SEM. Unpaired *t*-test; ****p < 0.0001.

To confirm that administration of venglustat formulated in a pelleted diet would produce adequate drug exposure and/or target engagement in plasma, brain, and CSF, we performed a 2-week treatment study in *Gba*^*D409V/WT*^ mice. Treated mice were fed venglustat formulated in a pelleted diet (0.03% wt/wt) and untreated littermates were fed a control diet for two weeks (Fig. 2A,B, C*left*). At necropsy, plasma, brain, and CSF were collected to evaluate drug quantities and/or glycosphingolipid levels. As a meaningful change in the accumulation of aggregated proteins in the CNS was unlikely to occur within such a short time frame, morphological assessment of hippocampal inclusions was not included as a study endpoint. CSF collected from *Gba*^*D409V/WT*^ mice was reserved for glycosphingolipid level analysis, as the very limited volumes of CSF obtained from these mice precluded us from also measuring drug concentrations in this biofluid. Adequate levels of exposure of this brain-penetrant GCS inhibitor was confirmed in plasma (1616 ± 221 ng of venglustat per mL of plasma) and brains (901 ± 110 ng of venglustat per g of brain lysate) of treated animals (Fig. 2C, *left*). Total GlcCer levels were quantified via mass spectrometry. Though *Gba*^*D409V/WT*^ mice did not accumulate GlcCer levels beyond that of their WT counterparts, animals treated with venglustat showed reduced levels of this glycosphingolipid in plasma, brain, and CSF (14 ± 2%, 68 ± 1% and 37 ± 3% of control *Gba*^*D409V/WT*^, respectively; *p* < 0.0001, *N* = 12) relative to untreated animals (Fig. 2C, *left*).

While we observed putatively beneficial outcomes using GCS inhibitors in *Gba*^*D409V/WT*^ mice, we next opted to evaluate the effects of long-term venglustat treatment in a more severe *Gba* model (Gaucher-related synucleinopathy mice, *Gba*^*D409V/D409V*^). These mice provide a more robust platform for evaluating the potential pharmacological benefits of GCS inhibitors. In addition to accumulating greater amounts of hippocampal protein aggregates and GlcSph, these mice develop cognitive impairment starting at approximately 4 months of age^22^. Our previous studies had validated GCS inhibition as a therapeutic strategy for synucleinopathies in *Gba*^*D409V/D409V*^ mice by demonstrating a significant improvement in CNS pathology and the associated memory deficit following treatment^27^. However, these results were achieved using GZ667161 and not the compound which has or is currently being evaluated in clinical trials for several disease indications, including GBA-PD^33^. As such, we sought to evaluate the effects of GCS inhibition in *Gba*^*D409V/D409V*^ mice using the clinical candidate compound, venglustat.

We first confirmed that the presence of an additional copy of mutant *Gba* did not negatively affect compound distribution or target engagement with a short-term treatment experiment. Here, four-month-old *Gba*^*D409V/D409V*^ mice were treated with venglustat in pelleted rodent chow (0.03% wt/wt) for two weeks prior to necropsy, where plasma, brain and CSF were collected. As a meaningful change in the accumulation of aggregated proteins in the CNS was unlikely to occur within such a short time frame, morphological assessment of hippocampal inclusions was not included as a study endpoint. The limited volumes of CSF collected from mice was reserved for measuring drug concentration. In treated *Gba*^*D409V/D409V*^ mice, venglustat concentrations were 1706 ± 51 ng/mL in plasma, 1184 ± 56 ng/g in the brain, and 56 ± 3 ng/mL in CSF (CSF data not shown)(Fig. 2C, *right*). Total GlcCer levels were quantified via mass spectrometry. As expected, *Gba*^*D409V/D409V*^ mice treated with venglustat had reduced levels of GlcCer in plasma and brain (10 ± 1% and 76 ± 2% of control *Gba*^*D409V/D409V*^, respectively; *p* < 0.0001, *N* ≥ 44) relative to untreated animals (Fig. 2C, *right*). Collectively, these data confirmed CNS exposure and GCS inhibition activity of venglustat in a mouse model of Gaucher-related synucleinopathy.

### Venglustat reduced glycosphingolipids in Gaucher-related synucleinopathy mice

**Figure 3:**
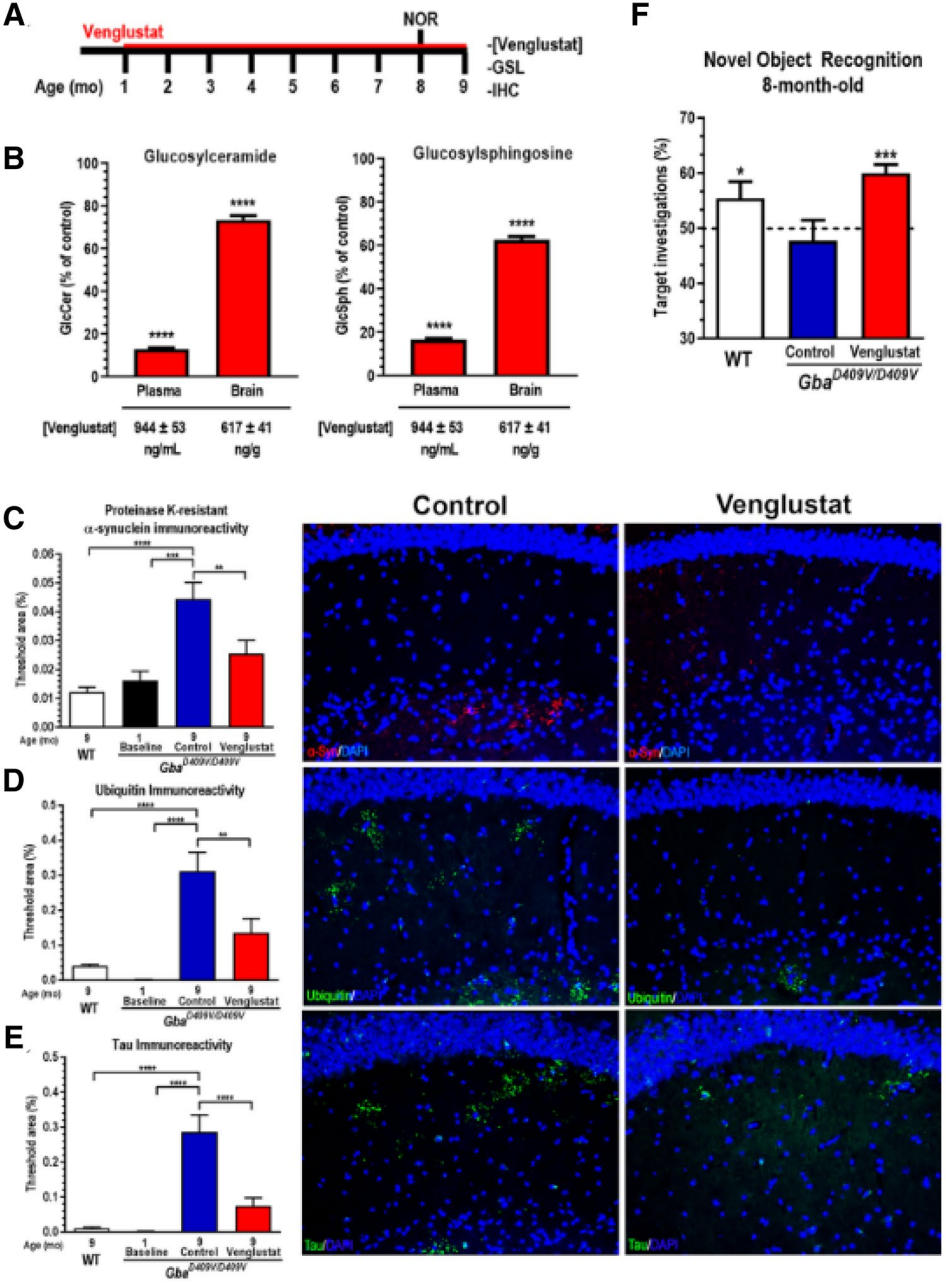
GCS inhibition by venglustat reduced glycosphingolipids and brain protein aggregate accumulations and improves the hippocampal-related memory deficit in a Gaucher-related synucleinopathy mouse model. (**A**) Timeline of sustained venglustat administration in Gaucher-related synucleinopathy mice (*Gba*^*D409V/D409V*^) and endpoints. Four-week-old animals were fed venglustat in pelleted diet (0.03% wt/wt) and control *Gba*^*D409V/D409V*^ mice were fed the same diet minus the drug as described in the Methods. Animals were euthanized after 8 months of treatment. NOR: novel object recognition behavioral assay; GSL: glycosphingolipid measurements; IHC: immunohistochemical analysis and quantification. (**B**) Venglustat significantly lowered GlcCer (*left*) and reduced GlcSph (*right*) accumulation in plasma and brain of treated *Gba*^*D409V/D409V*^ mice. Results are represented as the mean ± SEM, *N* ≥ 17 per group. Unpaired *t*-test; ****p < 0.0001. (**C**–**E**) Brain sections from WT and *Gba*^*D409V/D409V*^ mice were immunostained for proteinase K-resistant α-synuclein (**C**), ubiquitin (**D**), and tau (**E**) aggregates. Venglustat treatment significantly reduces the accumulation of aggregated proteins in the hippocampi of *Gba*^*D409V/D409V*^ mice. The representative images (*right*) demonstrate proteinase K-resistant α-synuclein immunoreactivity (**C**; red), ubiquitin immunoreactivity (**D**; green), and tau (**E**; green) of control and venglustat-treated *Gba*^*D409V/D409V*^ mice at 9 months of age. DAPI-stained cell nuclei are shown in blue. One-way ANOVA followed by Tukey’s post-hoc analysis. All data represent the mean ± SEM, with *N* = 9–20 per group; **p < 0.01; ***p < 0.001; ****p < 0.0001. (**F**) *Gba*^*D409V/D409V*^ mice were subjected to the novel object recognition test 7 months after treatment initiation. No groups exhibited preference during training when presented with two objects (data not shown). After 24 h, mice were presented with a novel object during the testing. WT mice showed significant preference for the novel object as indicated by an increase in the number of investigations of the novel object (*white bar*). *Gba*^*D409V/D409V*^ control mice (*blue bar*) showed no preference for the novel object, indicating cognitive impairment while venglustat treated *Gba*^*D409V/D409V*^ mice (*red bar*) demonstrated preference for the novel object as indicated by a significant increase in investigations of the novel target. Results demonstrate that venglustat treatment corrects the memory impairment in *Gba*^*D409V/D409V*^ mice. Column statistics with one-sample *t*-test. *p < 0.05 and ***p < 0.0001 signify means are significantly different than theoretical mean of 50%. *N* ≥ 17 per group.

To evaluate the effects of sustained GCS inhibition in this more robust synucleinopathy model, we performed a long-term treatment study using the pelleted chow formulation of venglustat in Gaucher-related synucleinopathy mice starting at 4 weeks of age (Fig. 3A). A control group of littermates was fed vehicle rodent chow. Brain exposure after 8 months of administration was 617 ± 41 ng/g (Fig. 3B), while plasma exposure was 944 ± 53 ng/mL (Fig. 3B). As anticipated, mice treated with venglustat for 8 months had significantly reduced levels of GlcCer relative to untreated animals, with 73 ± 2% remaining in the brain and 13 ± 1% remaining in the plasma compared to controls (Fig. 3B, *left*; *p* < 0.0001, *N* ≥ 17 per group; Suppl. Table 5). GlcSph, the accumulating glycosphingolipid substrate in this model, was even more substantially reduced in the CNS following venglustat administration, with 63 ± 2% remaining in the brain relative to untreated animals (Fig. 3B, *right*; *p* < 0.0001, *N* ≥ 17 per group; Suppl. Table 5). The reduction in plasma GlcSph was similar to that of GlcCer, with 17 ± 1% remaining compared to untreated animals (Fig. 3B, *right*; *p* < 0.0001, *N* ≥ 17 per group; Suppl. Table 5). These data are consistent with our previously reported reductions in glycosphingolipids in *Gba*^*D409V/D409V*^ mice treated with GZ667161^27^ and demonstrated the target engagement capability of the clinical candidate GCS inhibitor venglustat in this mouse model of Gaucher-related synucleinopathy.

### GCS inhibition by venglustat reduced the accumulation of aberrant protein aggregates in the hippocampus of Gaucher-related synucleinopathy mice

Gaucher-related synucleinopathy mice progressively accumulate neuronal inclusions composed of proteins including proteinase K-resistant α-synuclein, ubiquitin, and tau in the CNS^22,23^. In the present study, abnormal accumulations of these protein aggregates were absent in 9-month-old wild-type (WT) controls and 4-week-old *Gba*^*D409V/D409V*^ untreated baseline mice. In contrast, 9-month-old *Gba*^*D409V/D409V*^ controls had significant levels of hippocampal inclusions (Fig. 3C–E). Venglustat treatment reduced the amount of proteinase K-resistant α-synuclein, ubiquitin, and tau inclusions in *Gba*^*D409V/D409V*^ mice after 9 months of treatment (*P* = 0.009, 0.004, and < 0.0001, respectively; Fig. 3C–E). Collectively, these data support the hypothesis that restoration of lipid metabolism homeostasis by venglustat in mutant Gaucher mice slows or halts the accumulation of protein aggregates in the CNS. These findings are significant, as they further validated modulation of glycosphingolipid metabolism as a disease-modifying approach for treating Gaucher-related synucleinopathies.

### Venglustat administration prevented the development of the hippocampal-related memory deficit in Gaucher-related synucleinopathy mice

Mounting evidence implicates *GBA* mutations as a significant contributor to faster cognitive decline in patients with synucleinopathies, including PD^9–12^. In addition to the lipidopathy and proteinopathy phenotypes, Gaucher-related synucleinopathy mice also present with cognitive impairment^22,23^. To determine the effects of GCS inhibition by venglustat on cognition, hippocampal-related memory was evaluated with a novel object recognition test following 7 months of treatment. While WT mice preferentially investigated the novel object, untreated *Gba*^*D409V/D409V*^ mice showed no such preference, indicative of a memory deficit (Fig. 3F). Long-term treatment of *Gba*^*D409V/D409V*^ mice with venglustat attenuated these memory deficits as demonstrated by the preferential investigation of the novel object (*P* < 0.0001; Fig. 3F). These data demonstrated the ability of venglustat to improve this aberrant behavioral phenotype in a mouse model of Gaucher-related synucleinopathy.

## Discussion

Mounting evidence has strengthened the link between synucleinopathies, *GBA* mutations, and the glucocerebrosidase pathway and supports targeting the sphingolipid pathway as a potential therapeutic strategy for synucleinopathies. Mutations in *GBA* are the most significant risk factor for the development of synucleinopathies, acting to decrease the age of onset and accelerate disease progression^3,4,34,35^. Emerging evidence for reduced GCase activity in the CSF of idiopathic PD patients suggests that dysfunction in sphingolipid metabolism may even play a role in synucleinopathies beyond the genetically defined population (Parnetti et al., 2017). The present study supports the hypothesis that modulation of the sphingolipid pathway via inhibition of de novo GlcCer synthesis by brain-penetrant GCS inhibitors can ameliorate the proteinopathy and cognitive impairment phenotypes associated with synucleinopathies. Notably, these outcomes were achieved using venglustat, a brain-penetrant GCS inhibitor developed for clinical investigations.

The complexities of Parkinson’s disease pathogenesis cannot be replicated in a single preclinical model. In this study, we employed mouse models of synucleinopathy which vary in *Gba* mutation copy number and resultant GCase activity and exhibit a spectrum of synucleinopathy phenotypes^22^. Gaucher-related synucleinopathy mice (*Gba*^*D409V/D409V*^) have greatly reduced GCase activity (~ 25% of wild-type)^22^ relative to *GBA*-related synucleinopathy mice (*Gba*^*D409V/WT*^) (~ 50 of wild-type)^22^. As a consequence, they accumulate GlcSph and protein aggregates in the CNS to a greater extent and additionally develop a cognitive defect, a clinically relevant phenotype. While we observed beneficial outcomes in both *Gba* mouse models, the more robust behavioral and pathological phenotypes exhibited in the homozygous model allowed us to better evaluate the effects of venglustat on disease pathology. Of note, sustained administration of venglustat in *Gba*^*D409V/D409V*^ mice resulted in a significant improvement in memory impairment. These data are consistent with our previous work, which demonstrated an amelioration of cognitive dysfunction and even a reversal of the cognitive decline phenotype, following long-term administration of a tool compound GCS inhibitor (GZ667161) in *Gba*^*D409V/D409V*^ mice^27^. Importantly, the present study demonstrates the efficacy of the clinical candidate compound venglustat in ameliorating the aberrant cognitive phenotype in this mouse model of Gaucher-related synucleinopathy.

There are some technical limitations to evaluating substrate reduction therapy efficacy in the *GBA*-related synucleinopathy mouse model. Plasma GlcSph levels in *Gba*^*D409V/WT*^ mice could not be quantitated due to mass spectrometry assay sensitivity limits. Likewise, brain GlcSph levels were only detected in 4 out of 15 treated and 3 out of 10 control *Gba*^*D409V/WT*^ mice (Suppl. Table 1), limiting our statistical analysis. Future assay optimization experiments may improve GlcSph detection, expanding the dynamic range and permitting evaluation of subtle but potentially meaningful differences between treated and untreated heterozygous animals. A similar drawback exists for our assessment of insoluble CNS proteins, as *Gba*^*D409V/WT*^ mice accumulate fewer proteinaceous aggregates than *Gba*^*D409V/D409V*^ mice. A longer treatment duration and/or more sensitive aggregate detection methods may enable better differentiation between treated and untreated heterozygous animals. In addition, the lack of neurodegeneration in this mouse model precludes the assessment of whether GCS inhibition may provide neuroprotection.

There is a wealth of data supporting the hypothesis of a bidirectional pathogenic feedback loop between GCase and α-synuclein. Decreased GCase activity and the subsequent accumulation of undegraded glycosphingolipids (GSLs) can promote changes in membrane lipid homeostasis and the misprocessing of α-synuclein. In turn, increased intracellular accumulation of α-synuclein may prevent proper GCase trafficking from the endoplasmic reticulum to the Golgi apparatus and lysosomes. The resulting decrease in GCase activity in the lysosome can result in the accumulation of GSLs and exacerbate α-synuclein oligomerization^21^. In addition, reduced GCase activity may increase α-synuclein cell-to-cell transmission^24^ and reduce chaperone-mediated autophagy^20^, both of which can fuel neurodegeneration. This pathogenic cycle is linked to neuronal susceptibility, lysosomal dysfunction, and cognitive deficits. Remarkably, reducing the levels of GSLs via GCS inhibition reversed the toxic accumulation of aggregated α-synuclein in induced pluripotent (iPS) cell-derived neurons from Gaucher disease patients^30^. This hypothesis is further supported by the present studies, in which the reduction of GCase-related GSLs (by venglustat or GZ667161) yielded a numerical decrease in insoluble α-synuclein levels in the CNS of *Gba* mouse models. These data are consistent with our previous study, wherein GCS inhibitors (GZ667161) reduced the accumulation of these proteinaceous inclusions in the brains of *Gba*^*D409V/D409V*^ mice^27^. Similar beneficial outcomes have been demonstrated following AAV-mediated overexpression of human GCase in the CNS of *Gba* mutant homozygous mice; providing further validation that modulating GCase-related sphingolipid metabolism may be a viable therapeutic strategy for improving the proteinopathy and pathological phenotypes in synucleinopathies^23^.

Clinical trials have been completed or are currently underway to evaluate venglustat, an orally available, highly selective, brain-penetrant, small molecule GCS inhibitor, in several patient populations including chronic neuronopathic Gaucher disease (Gaucher disease type 3; NCT02843035), Fabry disease (NCT02228460), GM2 gangliosidosis (NCT04221451), autosomal-dominant polycystic kidney disease (NCT04705051), and early stage PD patients harboring heterozygous *GBA* mutations (NCT02906020), as a potential disease-modifying therapeutic. Despite the strength of these and other preclinical data, GCS inhibition by venglustat in early stage GBA-PD patients did not alter disease progression following one year of administration. Though clinical development for this indication has been halted, venglustat is still being pursued as a potential disease-modifying therapy in the additional rare disease indications (https://www.sanofi.com/en/media-room/press-releases/2021/2021-02-05-07-30-00). These differences between the clinical and preclinical results warrants further investigation to better understand the role of sphingolipids in Parkinson’s disease pathophysiology beyond their relationship with α-synuclein aggregation. Ongoing efforts to identify genetic modifiers of GBA-PD risk and age at onset, via genome-wide association studies, may aid in our understanding of these results^36^. Furthermore, such efforts may help to improve the design of future clinical studies by narrowing the selection of GBA-PD patients carrying particular variants, including those that are implicated in lysosomal dysfunction. Certainly, additional studies are required to determine the full impact of lysosomal dysfunction on PD pathogenesis. Yet, the ability to alter the course of disease through restoration of lipid metabolism indicate that the GCase-associated sphingolipid pathway is a key target for the development of transformative therapies for synucleinopathies.

## Materials and methods

### Animals

The Institutional Animal Care and Use Committee (IACUC) at Sanofi approved all procedures. All experiments were performed in accordance with relevant guidelines and regulations as stipulated by the IACUC. Animals were individually housed under light:dark (12:12 h) cycles and provided with food and water ad libitum. Environmental enrichment was provided in the form of corn cob bedding, nestlets, and cardboard nest boxes. Dietary enrichment was not provided to any groups. Mouse models of Gaucher disease, including Gaucher-related synucleinopathy (*Gba*^*D409V/D409V*^) and *GBA*-related synucleinopathy (*Gba*^*D409V/WT*^), harbor a point mutation at residue 409 in the mouse glucocerebrosidase (*Gba*) gene^22^ on a C57Bl/6 background. Age-matched C57Bl/6 wild-type (WT) mice, used as controls, were either littermates to *Gba*^*D409V/WT*^ mice or were purchased (Charles River Laboratories) for use in *Gba*^*D409V/D409V*^ studies. All behavioral testing was performed during the animals’ light cycle, between the hours of 8 AM and 4 PM. All methods and data are reported in accordance with ARRIVE guidelines (https://arriveguidelines.org) and recommendations.

### Administration of the glucosylceramide synthase inhibitors: venglustat and tool compound GZ667161

A subset of animals received glucosylceramide synthase inhibitors, venglustat (aka GZ402671) or GZ667161, via pelleted diet at 0.03%- or 0.033%-wt/wt, respectively. For each experiment, sex and siblings were randomly matched for group assignment. Target engagement and exposure confirmation studies included *Gba*^*D409V/D409V*^ or *Gba*^*D409V/WT*^ mice administered venglustat for two consecutive weeks beginning at approximately 4 months of age. Mice included in sustained GCS inhibition studies were administered either GZ667161 or venglustat upon weaning at ~ 4 weeks of age. Wild-type, baseline, and control groups were fed vehicle rodent chow. GCS inhibitor and vehicle diets were continuously provided to mice until necropsy and tissue collection.

### CSF collection

Animals were anesthetized via an intraperitoneal injection of a 10:1 Ketamine/Xylazine cocktail prior to being placed into a surgical ear bar rig. After making a midline cut to remove a small patch of skin from the head, the fat and muscle layers were opened using a cautery pen (Thermo Fisher Scientific; Waltham, MA) to reveal the base of the skull and occipital crest. The remaining tissue was then removed to expose the cisterna magna membrane. Using a pulled glass pipette (World Precision Instruments; Sarasota County, FL), the cisterna magna membrane was punctured to allow CSF to flow freely into the pipette via capillary action. After collecting approximately 10–20 uL, CSF was transferred to a clean protein lo-bind tube (Eppendorf; Hamburg, Germany). CSF samples with visible blood contamination were excluded from analyses.

### Animal perfusion and tissue and blood collection

Prior to whole blood collection, mice were anesthetized via a 200 uL intraperitoneal injection of sodium pentobarbital. Following the loss of response to a foot-pinch and corneal reflex, approximately 250 uL of whole blood was collected from the retro-orbital sinus using a glass capillary tube into a Microtainer® tube (BD Biosciences; Billerica, MA) containing K2 EDTA anticoagulant. Whole blood samples were collected retro-orbitally and immediately placed on ice. Plasma was isolated after 5 min centrifugation at 8000 RPM at 4 °C. Immediately following blood collection, animals were transcardially perfused with cold phosphate-buffered saline (PBS) at a rate of 18 mL/minute, for two minutes. After cutting the brains sagittally along the midline, the left hemisphere was microdissected into various regions, snap-frozen in liquid nitrogen, and stored at − 80 °C until use^27^. The right hemisphere was post-fixed in 10% neutral-buffered formalin for 48–72 h. Right hemispheres were then washed three times in 1X PBS and transferred to 30% sucrose for 24–48 h. Right hemispheres were embedded in O.C.T. and sectioned into 20 µm sections using a cryostat, as previously described^27^.

### Measurement of glycosphingolipid levels

Quantitative analysis of sphingolipids was performed by liquid chromatography and tandem mass spectrometry (LC–MS/MS)^28^. Briefly, brain tissue was homogenized in 10 volumes of water (w/v). Ten microliters of homogenate or plasma was extracted with 1 ml of extraction solution (50:50 acetonitrile/methanol) by protein precipitation. Mouse CSF sphingolipids were extracted by liquid–liquid extraction, as previously described^37^. GlcCer and galactosylceramide were separated using a Waters Acquity UPLC and Cortecs HILIC column (2.1 mm × 100 mm, 2.7 µm particles, Waters Corp., Milford, MA) and analyzed by an API 5000 triple quadrupole mass spectrometer in MRM mode (Applied Biosystems, Foster City, CA). GlcSph and psychosine were separated by a Waters Acquity UPLC and BEH HILIC column (2.1 mm × 100 mm, 1.7 µm particles, Waters Corp., Milford, MA) and analyzed by an API 6500 triple quadrupole mass spectrometer in MRM mode (Applied Biosystems, Foster City, CA). GlcCer and GlcSph standards were purchased from Matreya, LLC (State College, PA) and Avanti Polar Lipids (Alabaster, Al), respectively. All procedures were performed blinded to the genotype or treatment.

### Immunohistochemistry and morphometric analysis

To expose insoluble α-synuclein aggregates, some tissues were pretreated with proteinase K (1:4 dilution; Agilent, Santa Clara, CA) for 7 min at room temperature to digest soluble α-synuclein^23^. Brain sections were blocked with 10% (vol/vol) normal donkey serum for 1 h at room temperature and incubated with the following antibodies: mouse anti-ubiquitin (1:300; cat# MAB1510, Millipore; Burlington, MA), rabbit anti-α-synuclein (1:300; cat# S3062, Sigma), and mouse anti-tau (1:500, Tau-5; cat# MAB361, Millipore). Brain sections were then incubated for 1 h with either a donkey anti-mouse Alexa Fluor-488 (1:250 dilution, Invitrogen, Carlsbad, CA) or donkey anti-rabbit biotinylated secondary antibody (1:200 dilution; Jackson Immuno Labs; West Grove, PA). For α-synuclein aggregate quantification, a cyanine 3-tyramide signal amplification kit was used (PerkinElmer; Waltham, MA). Cell nuclei were counterstained with 4’, 6-diamino-2-phenylindole (DAPI) (Sigma-Aldrich, St. Louis, MO). Sections were coverslipped with aqua-poly/mount (Polysciences; Warrington, PA) and the stratum radiatum external to the CA1 hippocampal cell body layer was imaged with a SPOT camera (SPOT Imaging; Sterling Heights, MI) paired with a Nikon Eclipse E800 fluorescence microscope equipped with a 20 × objective lens, as previously described^22^. Two to three sections were imaged per animal and immunofluorescence was quantitatively measured via threshold fluorescent area on MetaMorph Software (Molecular Devices; San Jose, CA)^22^. All procedures were performed blinded to the treatment or genotype and the percent threshold area is expressed as the mean ± SEM^22^.

### Novel object recognition test

Testing was performed in a white Plexiglas open arena box measuring 20 × 20 × 10-high inches. Animals acclimated in the arena for 10 min prior to a 5 min inter-trial-interval in their home cage. Animals were then exposed to two symmetrically placed objects, A and B, for 5 min and the total number of target investigations was recorded. Animals that did not investigate each object a minimum of two times during these 5 min were disqualified from the test and excluded from analysis. After a 24-h retention period (day 2), non-disqualified animals were placed back into the arena and presented with a familiar object, A, and a novel object, C, for 5 min where the total number of target investigations were again recorded. Animals that did not investigate the novel object, C, a minimum of two times were disqualified from the test and excluded from the analysis. All procedures were performed unblinded to the genotype and treatment due to genotype-specific variations in the animals’ coat colors and the presence of a color-indicator in the formulated diets. Two animals, each from different treatment group, were tested simultaneously during each trial in separate arenas and scored by an independent investigator. Results are reported as the percentage of target investigations for the novel object during the testing trial on day 2. A target investigation score of 50% indicated no preference for either object.

### Statistical analyses

Parametric statistical analyses were performed either by Student’s *t*-test or analysis of variance (ANOVA) followed by Tukey’s post-hoc analysis. Nonparametric data were analyzed by Kruskal–Wallis test with Dunn’s multiple comparison analysis. Novel object recognition data was analyzed by column statistics and one-sample *t*-test to compare column means to a hypothetical value of 50% (50% signifies cognitive impairment using this behavioral test). All statistical analyses were performed using GraphPad Prism v4.0 (GraphPad Software; San Diego, CA). A *p-*value of < 0.05 was considered a statistically significant difference. Sample sizes were determined via a sample size calculation using historical means and standard deviations (obtained from the novel object recognition test), α = 0.05, β = 0.2, and a desired power of 0.80.

## Supplementary Information

Supplementary Tables.

## Data Availability

All data generated and/or analyzed during this study are included in this article and its Supplementary Information files.
