# Supplementary material for: Preclinical pharmacology of glucosylceramide synthase inhibitor venglustat in a GBA-related synucleinopathy model
Source: Sci Rep. 2021 Oct 22;11:20945. doi: 10.1038/s41598-021-00404-5 (PMC8536659; doi:10.1038/s41598-021-00404-5)
Supplement: Supplementary file 1 — Supplementary Tables. [file 41598_2021_404_MOESM1_ESM.docx]

**Supplementary Tables**


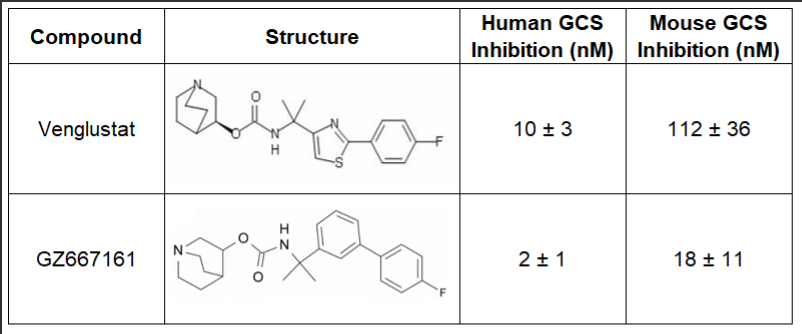


**Suppl. Table 1. GCS inhibition in human and mouse cell lines by venglustat and GZ667161.** The ability of venglustat and GZ667161 to inhibit GCS activity was explored following the conversion of labeled ceramide to glucosylceramide in human and mouse isolated microsomes as a source of enzyme. The IC_50_ for GZ667161 on this assay was 2 nM in human and 18 nM in mouse. In contrast, the IC_50_ for venglustat was 10 nM in human and 112 nM in mouse.


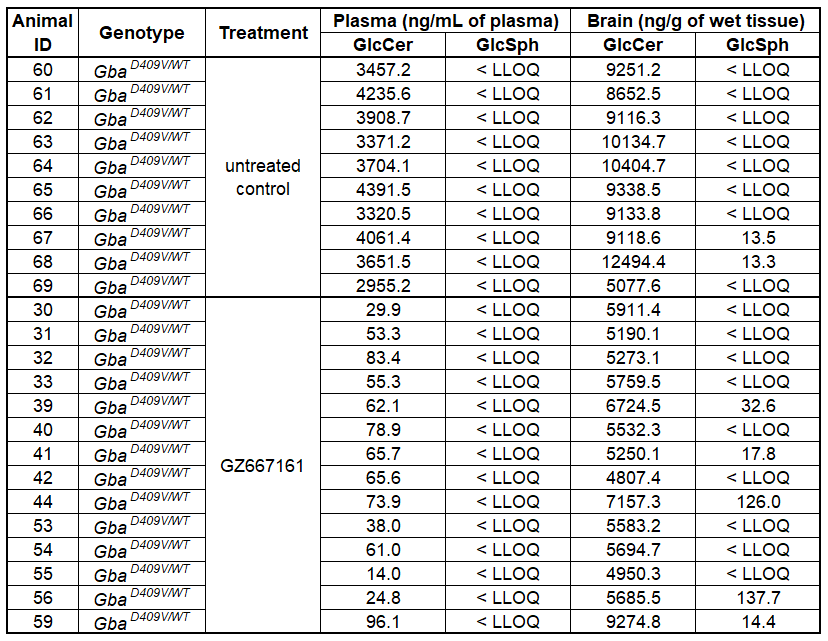


**Suppl. Table 2. GlcCer and GlcSph levels in brain and plasma of *GBA*-related synucleinopathy mice (*Gba^D409V/WT^*) treated with GZ667161 for 9 months.** GlcCer and GlcSph levels were quantitated via liquid chromatography tandem-mass spectrometry methods. Glycosphingolipid (GSL) measurements in plasma are represented as ng of GSL per mL of plasma, whereas GSL values in brain are denoted as ng of GSL per gram of wet brain tissue. < LLOQ: value was below the lower limit of quantitation (0.012 ug/g of brain tissue, 0.012 ng/mL of plasma) achievable by this method.


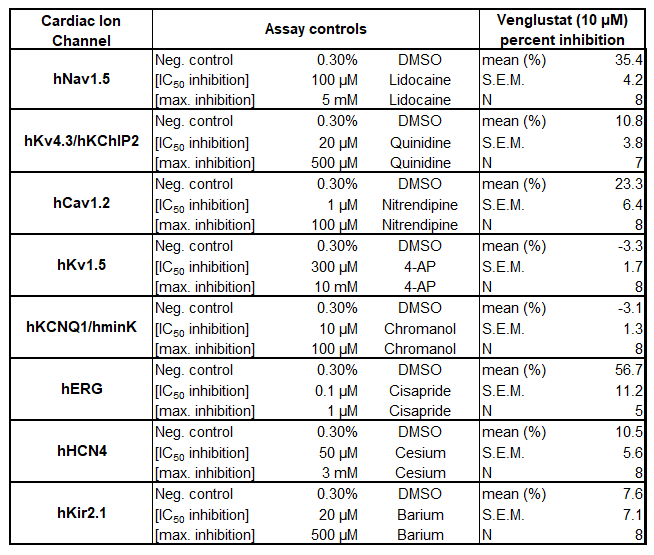


**Suppl. Table 3. Venglustat activity against cardiac ion channels.** Cardiotoxic effects of venglustat on various cardiac-related ion channel currents were evaluated using IonWorks™ Quattro (Millipore, Waltham, MA) in population patch mode. Venglustat’s effect at 10 µM on HCN4 currents was assessed via IonWorks™ HT (Millipore). DMSO (0.3%) served as the negative control while known ion channel inhibitors (Lidocaine, Quinidine, Nitrendipine, 4-AP, Chromanol 293B, Cisapride, Cesium, and Barium) were used as positive controls and tested at IC_50_ blocking and at a maximal blocking concentration. Data is represented as the percent mean inhibition by 10 µM venglustat for each ion channel.


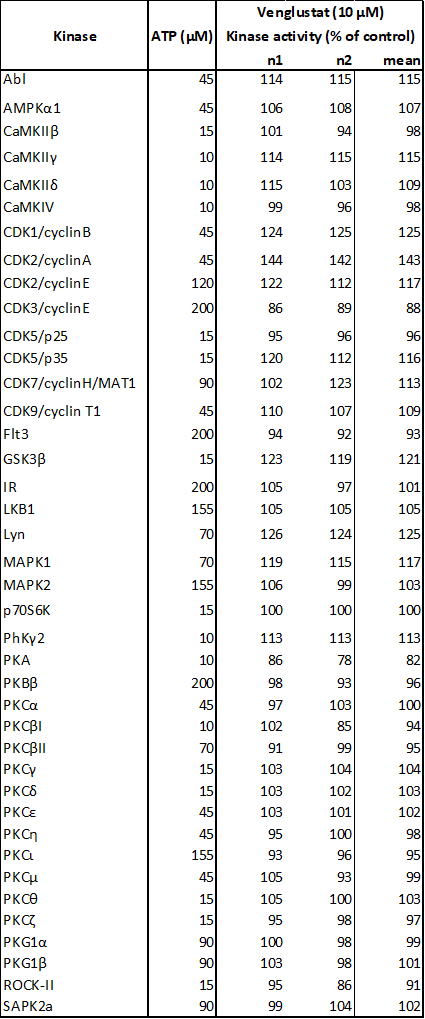


**Suppl. Table 4. Kinase activity profiling for venglustat.** The catalytic activity of kinases in the presence of 10 µM venglustat was assessed using a radiometric kinase activity assay (Millipore).

 **Suppl. Table 5. GlcCer and GlcSph levels in brain and plasma of Gaucher-related synucleinopathy mice (*Gba^D409V/D409V^*) treated with venglustat for 8 months.** GlcCer and GlcSph levels were quantitated via liquid chromatography tandem-mass spectrometry. Glycosphingolipid (GSL) measurements in plasma are represented as ng of GSL per mL of plasma, whereas GSL values in brain are denoted as ng of GSL per mg of total protein as assessed by a Pierce™ BCA protein assay (Thermo Fisher Scientific; Waltham, MA). < LLOQ: value was below the lower limit of quantitation (0.012 ug/g of brain tissue, 0.012 ng/mL of plasma) achievable by this method.
